# Rush Medical College

**Published:** 1871-11

**Authors:** 


### Rush Medical College.

We most cheerfully call the attention of our readers to the following appeal for Chicago. We believe it will meet a liberal response from not only the Alumni of the College, but from many others.

CHICAGO, November 1st, 1871.

*To Whom it may Concern :*

This is to certify, that the resident Alumni of Rush Medical College, of the City of Chicago, at a meeting held at the house of E. Ingals, M.D., on the eve of October 17, 1871, appointed the following Alumni an Executive Committee, to draft and present an appeal to the Alumni and friends of the College, for aid to rebuild and refurnish the College Building, viz.: T. D. Fitch, M.D., Chairman; H. A. Johnson, M.D., V. L. Hurlbut, M.D., C. T. Parkes, M.D., Ben C. Miller, M.D., and F. A. Emmons, M.D.

E. INGALS, M.D., Chairman.
CURTIS T. FENN, M.D., Secretary.

*An Appeal to the Alumni and Friends of the Rush Medical College, recently destroyed by fire, for aid to assist in its rebuilding :*

This College is among the oldest institutions of learning in the Northwest, having been in operation since 1843, at which time the region now tributary to Chicago was but sparsely populated, and had little wealth. During this time it has supplied a pressing need of this new country. It has educated a large number of young men, who are scattered through our whole country, worthily filling places of great usefulness and responsibility; and for this, both themselves and the public are indebted, in a great measure, to the school in which they received their instruction. A large proportion of its students have been possessed of little, save youth, hope, intelligence, and determination. Many of these, having been generously aided by the College, have taken rank among the most substantial members of the profession. The Faculty at all times, since its organization, has been moved by an earnest desire to promote the best interests of the profession and the College. For this its members have labored faithfully and earnestly; they have met the pecuniary burden of the School from its first foundation, and four years since they erected from their own resources, at an expense of $70,000, the most ample and best appointed college building on this continent, and filled it with every necessary appliance for successful teaching, and the influence and usefulness of the School has steadily increased from year to year. But in a day, the College Building, with all its contents, was swept away, along with a large part of the city, in which it stood a peer among many other noble institutions of learning. The pecuniary loss of the Faculty, in the destruction of the College, is light when weighed against others they have sustained. A number have lost nearly everything, and all have suffered much. The College must be rebuilt. Its past history, its future promise for good, demand no less. Under the circumstances, it is unreasonable to expect the Faculty to do this unaided. The College is now in a condition to justify an appeal to its Alumni, and to society, for some return for the favors it has conferred upon both. There is, perhaps, no field of benevolenee, that offers a richer return than to provide adequate and easy opportunities for instruction to those who desire to become learned in the best means for assuaging pain and healing the sick.

All donations may be remitted to Chas. T. Parkes, M.D., Elston Av., Chicago, who has been elected treasurer for the fund. They will be thankfully acknowledged, and faithfully devoted to the rebuilding of the College.

*The Trustees of Rush Medical College to its Alumni, Greeting :*

The last terrible conflagration which devastated so large and fair a portion of Chicago, swept out of existence nearly all the material part of your Alma Mater. Rush Medical College exists to-day only in its legal organization, the lot on which the College building stood, the energy of its Trustees and Faculty, and the love and fidelity of its Alumni.

The College edifice, so recently and expensively erected, the chemical and physiological laboratories, the museum, and all the appliances of teaching are gone and a sad material ruin replaces them.

The Trustees are, however, cheered and encouraged by the expressions of sympathy and offers of pecuniary assistance which have come to them from many of the Alumni, in different parts of the country. The Alumni of Chicago have appointed a committee, to appeal to their brethren, in behalf of their Alma Mater. This appeal the Trustees most heartily approve and endorse; and while all sums which may be offered will be most thankfully received, they are confident that fortune has smiled upon very many of the sons of " Old Rush," and that among these favored ones, there are generous hearts, which will prompt to munificent donations. To such they make the following offer:

For every donation of five hundred dollars the Trustees will establish a perpetual free scholarship, which shall bear the name of the donor, and which shall be conspicuously emblazoned on the wall of the lecture room. A certificate of this scholarship, engrossed on parchment, will be issued to the donor; which certificate shall secure to the bearer, free tuition, and when found qualified, free graduation. This certificate shall be perpetual in its operation; and thus the donor will have endowed for one student each year a Free Medical College.

WM. B. OGDEN, Chairman.
GRANT GOODRICH, Secretary.

